# Anti-Glycolytic Drugs in the Treatment of Hepatocellular Carcinoma: Systemic and Locoregional Options

**DOI:** 10.3390/curroncol30070485

**Published:** 2023-07-10

**Authors:** Miles Pourbaghi, Leila Haghani, Ken Zhao, Anita Karimi, Brett Marinelli, Joseph P. Erinjeri, Jean-Francois H. Geschwind, Hooman Yarmohammadi

**Affiliations:** 1 Department of Interventional Radiology, Memorial Sloan Kettering Cancer Center, New York, NY 10065, USA; pourbam@mskcc.org (M.P.); zhaok@mskcc.org (K.Z.); karimia@mskcc.org (A.K.); marinelb@mskcc.org (B.M.); erinjerj@mskcc.org (J.P.E.); 2 Oncology and Image-Guided Therapy, NAMSA, New York, NY 10021, USA; jfgeschwind@gmail.com

**Keywords:** hepatic artery embolization, hepatocellular carcinoma, Warburg effect, glycolysis, antiglycolytic drugs

## Abstract

Hepatocellular cancer (HCC) is the most common primary liver cancer and the third leading cause of cancer-related death. Locoregional therapies, including transarterial embolization (TAE: bland embolization), chemoembolization (TACE), and radioembolization, have demonstrated survival benefits when treating patients with unresectable HCC. TAE and TACE occlude the tumor’s arterial supply, causing hypoxia and nutritional deprivation and ultimately resulting in tumor necrosis. Embolization blocks the aerobic metabolic pathway. However, tumors, including HCC, use the “Warburg effect” and survive hypoxia from embolization. An adaptation to hypoxia through the Warburg effect, which was first described in 1956, is when the cancer cells switch to glycolysis even in the presence of oxygen. Hence, this is also known as aerobic glycolysis. In this article, the adaptation mechanisms of HCC, including glycolysis, are discussed, and anti-glycolytic treatments, including systemic and locoregional options that have been previously reported or have the potential to be utilized in the treatment of HCC, are reviewed.

## 1. Introduction

Hepatocellular carcinoma (HCC) is the most common primary liver cancer and is the third leading cause of cancer-related death [1]. (Liver transplant, surgical resection, and ablation are among the curative treatment options [2]. However, most patients are not candidates for curative options at the time of their diagnosis. These patients are treated with other options, including locoregional therapy, i.e., trans-arterial embolization (TAE or bland embolization), transarterial chemoembolization (TACE), transarterial radioembolization (TARE) and hepatic artery infusion (HAI). TAE and TACE can achieve local tumor growth control and improve overall survival (OS) in patients with unresectable HCC [3]. HCC is a hypervascular tumor that relies predominately (85%) on the hepatic artery. By contrast, the liver parenchyma relies primarily (75%) on the portal vein. TAE and TACE occlude the hepatic artery with embolic material causing ischemia and depriving the tumor of essential nutrients. Despite the high success rate reported with TAE and TACE, the overall survival is limited to 2 years, and the recurrence rate remains high [3,4]. Therefore, there is an urgent need to improve the efficacy of currently available intra-arterial therapies.

Hypoxia blocks the oxidative phosphorylation metabolic pathway and can lead to the arrest of proliferation and tumor necrosis. However, the explant evaluation of tumors treated with TAE/TACE shows that only a limited number of lesions demonstrate complete necrosis, indicating that these tumors are resistant to hypoxia and have adapted to hypoxia [5].

HCC uses multiple adaptive mechanisms to survive hypoxia and nutritional deprivation. One of these mechanisms is conversion to a high glycolytic state even in the presence of oxygen, also known as the “Warburg effect” [6]. Indeed, in 1956, Otto Warburg hypothesized that cancer cells consume glucose and produce lactate at a significantly higher rate compared to non-cancerous resting cells. Cancer cells, including HCC cells, even in a normoxic environment and in the presence of oxygen, rely on glycolysis for energy production and growth. Through this adaptation, cancer cells, including HCC, manage to survive hypoxia and sustain a high growth/proliferation rate [7].

Recently, there has been significant progress in the understanding of the “Warburg effect”, resulting in the development of several drugs that target glycolysis. In this review, these drugs are presented, and in vitro/in vivo studies are discussed.

## 2. Warburg Effect

Normal resting or differentiated cells rely on oxidative phosphorylation for producing energy in the form of adenosine triphosphate (ATP). During oxidative phosphorylation, which occurs in the mitochondria, these cells use pyruvate to form acetyl-CoA. Acetyl-CoA reacts with oxaloacetate in the tricarboxylic acid (TCA) cycle, also known as the Krebs cycle, to form carbon dioxide. The product of the complete oxidation of one glucose molecule is 38 ATP molecules [8]. The Krebs cycle happens only in the presence of oxygen. In hypoxic environments, normal cells convert pyruvate to lactate, producing 2 mol ATP per mol glucose. This is also known as anaerobic glycolysis and results in lactic acidosis. In 1956, Otto Warburg, a German scientist, and Nobel Prize winner, suggested that cancer cells use a different metabolic pathway. He postulated that cancer cells are in a hyperglycolytic state and upregulate glucose uptake. They consume glucose and produce lactate even in the presence of oxygen, producing approximately 4 mol ATP/mol glucose [6]. This phenomenon was named the “Warburg effect”, also known as aerobic glycolysis. Cancer cells are adapted to perform this cycle rapidly, resulting in enough energy for rapid growth, proliferation, and metastasis.

Anaerobic glycolysis is an inefficient method of producing energy with only 2 ATP per glucose molecule. Therefore, oxidative phosphorylation, which produces 36 ATP molecules per glucose molecule, is the preferred method of metabolism in the majority of normal cells. Similar to anaerobic glycolysis, one might assume that aerobic glycolysis (Warburg effect) is also inefficient. However, studies have demonstrated that the process of aerobic glycolysis is extremely efficient and faster than anaerobic glycolysis [9]. Therefore, a large number of ATPs are produced, and therefore, aerobic glycolysis is able to meet the high demand for energy in an aggressive, rapidly growing malignant cell. Consequently, due to their adaptation to undergo this cycle at a rapid pace, cancer cells generate sufficient energy for accelerated growth, proliferation, and metastasis. The heightened metabolic rate of cancer cells through glycolysis provides crucial resources, such as pyruvate, which is an essential resource for rapid growth in cancer cells. Additionally, the hyperglycemic state of cancer cells allows other metabolic pathways, including the pentose phosphate pathway, to become involved.

HCC cells, similar to other aggressive cancer cells, overexpress multiple enzymes that are involved in glycolysis [10]. Therefore, HCC demonstrates an escalated high rate of glycolysis. Normal liver cells depend on the glucokinase enzyme (type IV hexokinase). However, unlike normal liver cells, HCC cells considerably express another enzyme known as hexokinase type II (HK II) and down-regulate glucokinase. Furthermore, during the malignant transformation of HCC cells, other glycolytic enzymes, including glyceraldehyde-3 phosphate dehydrogenase (GAPDH) and lactate dehydrogenase (LDH), are also up-regulated. The difference in the rate of glycolysis in normal and cancer cells, in which cancer cells demonstrate a much higher glucose uptake, has been utilized as a diagnostic tool in positron emission tomography (PET). A glucose analog known as fluorodeoxyglucose can be utilized for tumor imaging and PET-guided procedures.

Since cancer cells heavily depend on glycolysis as their primary pathway for ATP production, targeting glycolysis and inhibiting glycolysis would significantly hamper ATP production in cancer cells and could potentially result in the selective destruction of cancer cells while sparing the normal surrounding tissue.

## 3. HCC Microenvironment and Adapting Mechanisms

HCC commonly develops in the cirrhotic liver. Chronic liver injury causes the replacement of normal liver parenchyma by fibrosis, demolishing the normal liver’s architecture and ultimately causing liver cirrhosis. Fibrotic changes damage the blood supply in the liver and consequently lead to chronic hypoxia in the cirrhotic liver. Hypoxia activates hypoxia-induced factor-1 (HIF-1), which is a major transcription factor and plays an essential role in the adaptation of HCC to hypoxia [10]. HIF-1 is made of two units, HIF-1α and HIF-1β, which is also known as the aryl hydrocarbon receptor nuclear translocator (ARNT) [10]. In the presence of oxygen (normoxia), HIF-1α was degraded by a proteasome. However, in hypoxia, this proteasome was inactive, and HIF-1α was stabilized and bound with HIF-1β, which translocated to the nucleus to form HIF-1. HIF-1 then induced the expression of multiple genes that resulted in angiogenesis, increasing glycolysis, and increasing lactic acid production. Hypoxia results in the stimulation and increase in the production of multiple growth factors, including the vascular endothelial growth factor (VEGF), platelet-derived growth factor (PDGF), and insulin-like growth factor (IGF). These growth factors stimulate angiogenesis, resulting in endothelial cell proliferation [11,12]. Additionally, HIF-1 upregulates glucose transporters, including GLUT 1, and upregulates key glycolysis enzymes, including HK II, LDH, and GAPDH, by 90% [13,14]. Upregulated glycolysis produces large amounts of lactic acid, which results in an acidic microenvironment. This highly acidic environment negatively impacts anti-tumoral characteristics and kills T-cell activities [15].

HCC has three histologic types: well, moderately, and poorly differentiated [16]. Previous studies have demonstrated that as HCC becomes less differentiated, histologically more cancer cells convert to aerobic glycolysis [17]. Poorly differentiated HCC has the propensity to exhibit venous invasion, either portal or hepatic, and is associated with a worse prognosis [18,19,20]. In summary, the more that cancer cells become dedifferentiated, the more they rely on glycolysis, and the more aggressive the cancer becomes, with a higher risk of venous invasion and a worse prognosis.

## 4. Effect of Arterial Embolization on HCC Microenvironment

Arterial embolization results in hypoxia. Hypoxia inactivates the oxidative phosphorylation pathway and causes mitochondrial dysfunction, which results in an increase in glycolysis, and an increase in cytoplasmic lactic acidosis, leading to an acidotic microenvironment. Lactic acidosis significantly reduces the rate of glycolysis, and thus, a small amount of glucose can support cancer cells for a long time. Therefore, lactic acidosis protects HCC from glucose starvation. Hypoxia stimulates the synthesis of VGEF, PDGF, and IGF [11,12]. Severe ischemia also induces quiescence in survived HCC cells and promotes cellular reliance on autophagy [21]. Therefore, hypoxia plays a double role in the treatment of HCC, causing tumor necrosis in a majority of the treated tumor and promoting tumor adaption to hypoxia in residual viable cells.

## 5. Targeting Glycolysis

**Figure 1:**
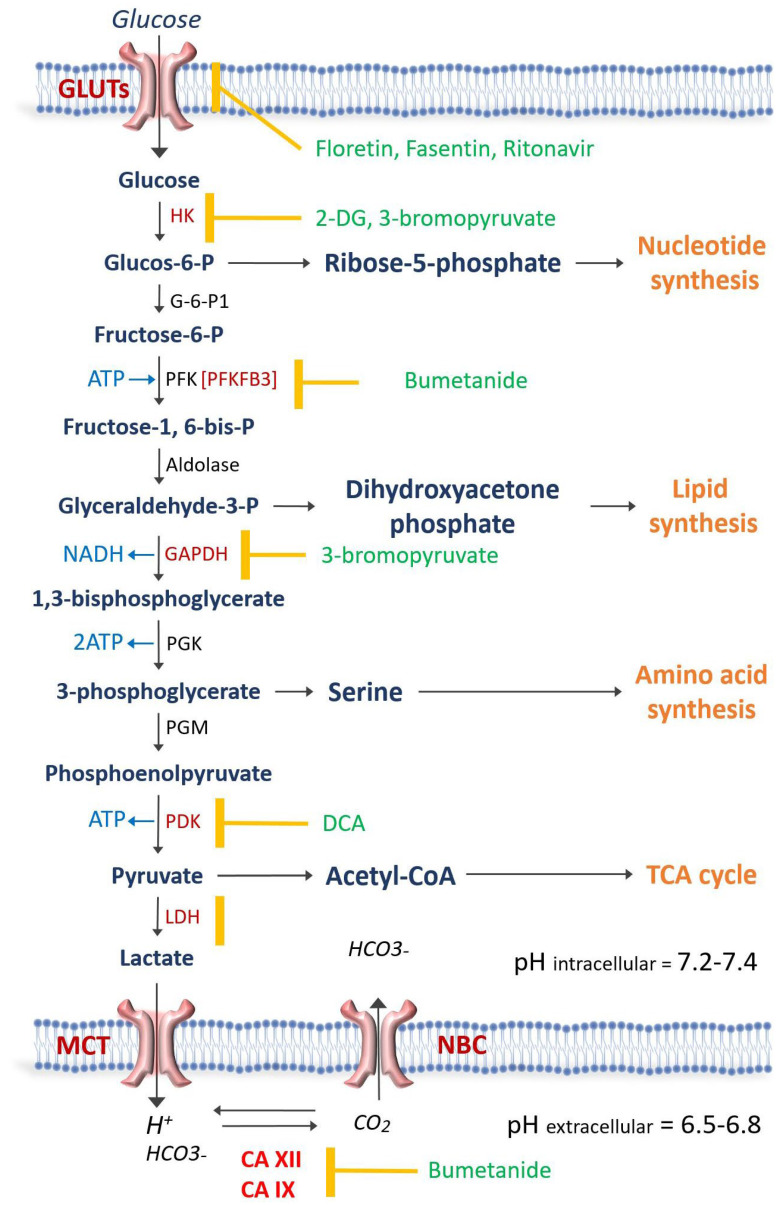
Schematic representation of glycolysis and then the enzyme involved. The antiglycolytic drugs are shown in green and the enzyme or transporter that each drug blocks is demonstrated by the yellow mark. Carbonic anhydrases XII (CA XII) and CAIX regulate the intracellular pH at 7.2–7.4 in order for the glycolytic enzymes to be active. Na-Bicarbonate cotransporter (NBC) and Monocarboxylate transporters (MCT) play a crucial role in pH regulation. GLUT = Glucose transporter; hexokinase = HK; G-6-P1 = Glucose-6 phosphate dehydrogenase; PFK = Phosphofructokinase; GAPDH = glyceraldehyde phosphate dehydrogenase; PGK = phosphoglycerate kinase; PGM = phosphoglycerate mutase; PDK = pyruvate dehydrogenase kinase; LDH = Lactate dehydrogenase; 2-DG = 2-deoxy-D-glucose; DCA = Dichloroacetate; TCA = Tricarboxylic acid.

As discussed above, cancer cells upregulate glycolysis before becoming extremely dependent on glucose and sensitive to changes in glucose concentration levels. A better understanding of the mechanisms underlying tumor adaptation mechanisms, such as its high reliance on glycolysis, has resulted in the development of several promising anti-glycolytic drugs [22]. Two therapeutic areas have been targeted: glucose uptake and the enzymes that are specifically involved in glycolysis (Figure 1 and Table 1) [23]. Figure 1 illustrates the steps involved in glycolysis and corresponding anti-glycolytic drugs. Table 1 demonstrates a list of anti-glycolytic drugs, their mechanisms of action, and the cancers that these drugs have been used to treat.

## 6. Targeting Glucose Transporters

Glucose transporters, including GLUT-1 through 12, are located on the surface of almost all cell types, whether malignant or normal [46]. The goal of targeting these transporters is to deprive the cancer cells of glucose [47]. This group includes GLUT-1 antibodies, ritonavir, BAY-876, fasentin, phloretin, silybin, quercetin, and Sodium-Glucose Linked Transporter.

### 6.1. GLUT-1 Antibody

*GLUT-1 antibody* blocks GLUT-1. GLUT-1 is expressed in several malignancies, including breast, lung (non-small cell lung cancer = NSCLC), and head and neck cancers [48]. Rostagi et al. demonstrated that anti-GLUT-1 antibodies could inhibit the proliferation of NSCLC and breast cancer cell lines by 50% and 75%, respectively, and induce apoptosis [24]. These antibodies could increase the cytotoxic effect of multiple chemotherapeutics, including cisplatin, paclitaxel, and gefitinib.

### 6.2. Ritonavir

Ritonavir, a protease inhibitor used in the treatment of HIV and more recently in the treatment of COVID-19 (in the form of PAXLOVID = ritonavir plus nirmatrelvir), blocks GLUT-4 [25]. In vivo and in vitro studies have demonstrated that ritonavir has a cytotoxic effect on multiple myeloma, breast, ovarian, and melanoma cancer cell lines [25].

### 6.3. BAY-876

BAY-876 is an antagonist of the GLUT1 receptor. This small molecule is a yellow-colored lipophilic powder that can be administered orally. It exhibits potent inhibitory effects on GLUT1, and its inhibitory concentration 50 (IC50) value in a cell-free system is as low as 2 nmol/L. Nonetheless, the clinical use of BAY-876 has been confronted with numerous obstacles [49]. Advanced HCC patients showed increased GLUT1 expression, worsening their prognosis, which BAY-876 could help mitigate.

Primarily, many HCC patients suffer from severe cirrhosis, leading to compromised gastrointestinal function. This, in turn, results in these patients experiencing suboptimal absorption of orally administered medications [50]. Secondly, the systemic distribution of the drug caused by oral consumption or intravenous infusion can disrupt the body’s natural glucose uptake process. Additionally, it can also result in an insufficient concentration of BAY-876 reaching the lesion. To address these obstacles, Yang et al. formulated a microcrystalline version of BAY-876 and tested its sustained release and antitumor activity in HCC models [26]. Injecting microcrystalline BAY-876 directly into HCC tissues can lead to localized drug presence, inhibiting glucose uptake, cell proliferation, and the expression of EMT-related factors. This localization restricts the spread to other tissues, thus minimizing possible adverse effects. This offers a promising new option for advanced HCC treatment.

### 6.4. Fasentin

Fasentin is a small-molecule *N*-[4-chloro-3-(trifluoromethyl) phenyl]-3-oxobutanamide that blocks GLUT-1 by interacting with a specific intracellular channel portion of it [51]. Limited data are available on its cytotoxic effect.

### 6.5. Phloretin, Silybin, and Quercetin

Phloretin, 2′,4′,6′-trihydroxy-3-(4-hydroxyphenyl)-propiophenone, silybin, and quercetin are Flavanoids that are extracted from natural products [52]. Phloretin is a natural phenol that is extracted from apple tree leaves or apricot. Phloretin blocks GLUT 2 and causes apoptosis in HCC cells [27]. Phloretin also enhances the cytotoxic effects of paclitaxel in HCC cancer cells [28]. An in vivo study demonstrated that silybin, another natural GLUT blocker, was able to inhibit growth in HCC [29]. Quercetin blocks GLUT-1, and a recent study on HCC cell lines has shown that it can induce apoptosis in HCC [30].

### 6.6. Sodium-Glucose Linked Transporters

Sodium-glucose-linked transporters (SGLTs) are proteins that facilitate the transport of glucose across cell membranes. There are two major types of SGLTs in humans: SGLT1 and SGLT2. Both SGLT1 and SGLT2 are found in the kidney, but SGLT2 is primarily responsible for reabsorbing filtered glucose from the urine back into the bloodstream. This reabsorption process is energy-efficient because it is coupled with the reabsorption of sodium ions, which flow down from their concentration gradient [53]. SGLT2 inhibitors are oral medications that help lower blood sugar in individuals with type 2 diabetes by inhibiting glucose reabsorption within the kidneys, which causes the body to excrete more sugar in the urine [54]. Currently, there are four SGLT2 inhibitors that are commercially accessible in the United States, including canagliflozin, dapagliflozin, ertugliflozin, and empagliflozin.

A growing body of preclinical and clinical trials has suggested that SGLT2 inhibitors could potentially enhance the condition of Non-Alcoholic Fatty Liver Disease (NAFLD) [54,55]. It has been numerously reported that SGLT2 inhibitors could potentially have anti-cancer properties. These molecules block the uptake of glucose in cancer cells, which can lead to the death of these cells [56]. Kaji et al. demonstrated that the SGLT2 inhibitor canagliflozin directly inhibited the proliferation of cells in a mice-bearing liver tumor model [31]. This study found that canagliflozin reduced the growth of liver cancer cells by blocking their uptake of glucose. In another study, Hendryx et al. conducted an extensive study using SEER-Medicare linked data and demonstrated that the initiation of SGLT2 inhibitors appeared to enhance overall survival in HCC patients who already had type 2 diabetes compared to those not using SGLT2 inhibitors [57]. Correspondingly, other experimental studies have also hinted at the possible advantage of SGLT2 inhibitors when treating liver cancer [58].

## 7. Targeting Enzymes Involved in Glycolysis

Glycolysis involves multiple enzymes, including hexokinase, glyceraldehyde 3-phosphate dehydrogenase (GAPDH), lactate dehydrogenase (LDH), pyruvate dehydrogenase kinase (PHK), and phosphofructokinase (PFK) [59].

### 7.1. 2-Deoxy-D-Glucose (2-DG)

2-deoxy-D-glucose (2-DG) is a glucose analog that has demonstrated promising effects as a potential therapeutic agent in treating cancer. 2-DG competitively inhibits glucose uptake via GLUT1 and also disrupts hexokinase, which is the initial and rate-limiting step of glycolysis [60]. 2-DG is converted to 2-DG-phosphate, which cannot leave the cell, and its accumulation in the cell is attributed to ATP depletion and oxidative stress, ultimately resulting in cellular death [32,33].

The observations of reduced cellular proliferation and induction of metabolic arrest have been documented in various cancer cell types, including HCC, following treatment with 2-DG [61,62,63]. An in vitro study has shown that 2-DG was able to induce apoptosis in HepG2 and Hep3B HCC cell lines [63]. Based on in vivo studies, the daily administration of 2-DG can lead to hypoglycemia-like symptoms, thereby constituting one of the main limitations of 2-DG as a monotherapy [64,65].

Oral dosing has been administered to treat prostate cancer cells and cerebral glioma [34,35,36]. Several studies have demonstrated that 2-DG might not be a good candidate for monotherapy. However, when used in combination with other chemotherapeutic drugs, such as docetaxel, the combination of these drugs was well-tolerated, and a synergistic effect was observed [34]. Another potential application of 2-DG is a radiosensitizer, which was used in combination with radiation therapy. An oral daily dose of 2-DG was combined with external beam radiation to treat patients with brain tumors [35,36]. Patients with Glioblastoma multiforme and anaplastic astrocytoma were treated with 250 mg/kg of 2-DG once a week and 5 Gy of external beam radiation for 7 weeks. The 250 mg/kg appeared to be the maximum tolerable dose, and higher doses resulted in side effects. Radiation combined with 2-DG was able to enhance tumor necrosis while the normal brain was relatively protected [66,67].

### 7.2. Dichloroacetate (DCA)

Dichloroacetate (DCA) inhibits pyruvate dehydrogenase kinase (PDK). By inhibiting PDK, DCA has been shown to disinhibit pyruvate dehydrogenase (PDH), which resulted in increased conversion of pyruvate to acetyl-CoA, ultimately reversing the glycolytic phenotype and switching cells from glycolytic pathways to oxidative phosphorylation [37].

DCA has been investigated in the context of congenital disorders associated with mitochondrial dysfunction. DCA has been beneficial to these patients by redirecting cellular metabolism from glycolysis to oxidative phosphorylation and consequently reducing lactate production [68].

Since DCA is theoretically capable of reversing the Warburg effect, DCA has been considered a promising candidate as an antiglycolytic agent in the treatment of numerous tumor cells, including breast, prostate, medullary thyroid, lung, myeloma, endometrial and the glioblastoma multiforme [37,38,39,40,41,42,69]. A dose-escalation study involving 24 patients with solid tumor malignancies who received DCA as a monotherapy revealed the occurrence of adverse effects such as fatigue, nausea, and neuropathy [70,71]. However, when this dosage was lowered to 6.25 mg/kg twice daily, adverse effects were notably reduced. Despite this, no RECIST-defined response was observed [70,71].

The efficacy of monotherapy with DCA remains uncertain. In vitro and in vivo monotherapy has shown mixed results. However, when combined with other drugs, promising results were reported [70]. When combined with sorafenib, a tyrosine-kinase inhibitor, DCA demonstrated a reduction in the development of sorafenib resistance in a subcutaneous xenograft HCC mouse model [70]. In another study, DCA combined with doxorubicin was used to treat LM3 hepatoma mice [72]. DCA was able to enhance the efficacy of doxorubicin, and the combination of DCA and doxorubicin was able to significantly reduce tumor growth.

Doxorubicin might be limited by its cytotoxicity to cardiomyocytes and hepatocytes. Therefore, combining it with DCA allowed lower dosing to be administered by exploiting the inherent metabolic and apoptotic effects of DCA, leading to an increase in doxorubicin cytotoxicity without any increase in unwanted toxicities [72].

### 7.3. 3-Bromopyruvate (3-BrPA)

The resurgence of research interests in tumor metabolism, coupled with an enhanced understanding of the molecular mechanisms governing tumor glycolysis, has encouraged the development of agents that specifically target glycolysis. While several of these agents have been examined for their therapeutic potential in preclinical tumor models, most have not progressed to clinical trials due to inadequate efficacy and significant toxicities. However, one metabolic inhibitor called 3-bromopyruvate (3-BrPA), which is a halogenated derivative of pyruvic acid, has recently garnered significant attention due to its remarkable antitumor effects and low toxicity profile. By introducing a bromine atom to pyruvate, 3-BrPA possesses alkylating properties, allowing it to form an irreversible chemical bond with its target [73,74].

In vitro experiments conducted on human HCC cells demonstrated that 3-BrPA effectively suppressed glycolysis and impeded ATP production, leading to apoptosis in a manner dependent on the dosage administered. Further investigation using radiolabeled 3-BrPA revealed that the key intracellular target of this agent was GAPDH. GAPDH was overexpressed in neoplastic cells and catalyzed the sixth step of glycolysis. GAPDH was directly inhibited by the binding of 3-BrPA, thereby disrupting the enzyme’s activity and ultimately hindering glycolytic ATP production, resulting in apoptotic cell death.

Studies have demonstrated that GAPDH plays a significant role in tumorigenesis and chemoresistance [43,44]. Once GAPDH was blocked via 3-BrPA, = massive depletion of intracellular ATP developed, resulting in apoptosis [74]. In addition, the uptake of 3-BrPA into cancer cells depended on MCT-1 transporters. Because these transporters were upregulated in neoplastic cells, 3-BrPA selectively targeted cancer cells with a higher number of MCT-1 transporters [75]. As a result, 3-BrPA has the potential to be an extremely promising anti-cancer agent.

In contrast to other alkylating agents, 3-BrPA exhibits remarkable specificity in its molecular targeting, leading to its potent anti-tumorigenic effects by simultaneously causing energy depletion, disrupting redox balance, and inducing intracellular stress. This multifaceted approach makes 3-BrPA an exceptionally promising agent due to its selectivity towards tumor cells and its ability to exert a comprehensive antitumor impact.

Both in vitro and in vivo investigations have consistently demonstrated the impressive efficacy of 3-BrPA in eliminating various types of cancer cells, including HCC [76]. In the treatment of liver cancer, image-guided procedures, particularly intra-arterial therapies, hold significant importance. These locoregional approaches offer advantages by providing access not only to the core but also to the periphery of the tumor. Furthermore, they allow for the attainment of higher drug concentrations within the tumor while minimizing systemic exposure. Consequently, the intra-arterial delivery of 3-BrPA has been evaluated in different animal models of liver cancer, yielding highly encouraging results. The tumors were effectively eradicated, often leading to the substantial prolongation of survival, and some animals even achieved a complete cure despite the aggressive nature of these tumors.

Numerous studies have demonstrated the efficacy of this drug when given locally under image guidance [77,78,79,80,81]. In one study, 3-BrPA was administered intra-arterially in 32 VX2 liver tumor-bearing rabbits and demonstrated complete tumor necrosis with no additional harm to the liver [78]. However, at higher doses of this drug, beyond the maximum therapeutic dose (MTD), some degree of peripheral liver necrosis was found [78]. Finally, when the drug was microencapsulated to protect it as it navigated through the systemic circulation, it was found to completely eradicate pancreas cancer in nude mice providing the most promising proof yet of high anti-cancer efficacy combined with an extremely favorable toxicity profile [81].

Combined therapies with 3-BrPA have also shown promising results [82], especially when combined with cisplatin and oxaliplatin to treat colon cancer cell lines. Such a combination of anticancer drugs offers the potential for dose reduction and overcoming resistance [82].

### 7.4. Bumetanide = (BU)

Bumetanide (BU) is a loop diuretic with a rapid onset and short duration of action. BU interferes with renal cAMP and/or inhibits the sodium-potassium ATPase pump. It also blocks the active reabsorption of chloride [83]. BU blocks both carbonic anhydrase IX (CAIX) and XII (CAXII) and blocks the transport of chloride into cancer cells, ultimately leading to the decreased transport of HCO_3_- into these cells [84,85]. Therefore, the intracellular pH decreases, making the internal milieu acidic and blocking the rate-limiting step of phosphofructose kinase (PFK1). As described earlier, once the cancer cells shift to aerobic glycolysis, large amounts of lactic acid accumulate in the cytoplasm, which decreases the intracellular pH to a level that is no longer tolerable [86]. However, HCC and other cancer cells overexpress CAIX and CAXII in which bicarbonate ions are imported while lactate is exported, leading to decreases in the extracellular pH and increases in the intracellular pH to an acceptable level [87,88]. BU especially decreases the intracellular pH in cells that overexpress CAIX and CAXII, leading to the inhibition of PFK1, which is the most sensitive to pH glycolytic enzymes, blocking glycolysis. In 1977, Lubowitz studied human red blood cells and demonstrated that BU has anti-glycolytic effects [89].

BU demonstrated significantly enhanced tumor necrosis in an N1S1 HCC tumor model in rats [45]. This study was performed on 14 rats that were divided into three groups: a control group, hepatic artery embolization group (TAE), and the combined BU plus TAE group. The tumors in the combined TAE plus BU group showed a 90.4 ± 10.2% decrease in size, which was a 72.2% greater size decrease when compared to the TAE-only group. This study has led to the initiation of an ongoing phase I/II trial (ClinicalTrials.gov Identifier: NCT03107416) in patients with unresectable HCC, in which patients are being treated with a combination of TAE plus intra-arterial BU.

## 8. Discussion/Conclusions

Glycolysis is an important part of cancer adaption and resistance to the available locoregional therapies for HCC. The monotherapy of available antiglycolytic drugs has not delivered promising results when observed in both in vitro and in vivo studies. Additionally, the effective systemic dose of these drugs has, at times, been associated with severe side effects [90]. Prior experience has shown that the locoregional delivery of these drugs has allowed us to deliver the drug in high doses without having systemic toxic effects. Additionally, combining these drugs with other treatment options has been shown to improve efficacy and allow the usage of lower doses [72,82]. Finally, some anti-glycolytic drugs have been shown to increase the sensitivity of these tumors to radiation.

In summary, the combination of antiglycolytic drugs with available locoregional options, including TAE, TACE, and radioembolization, has the potential to reduce the toxicity of chemotherapy or radiation and improve the efficacy of available treatment options.

## Figures and Tables

**Table 1:** The list of antiglycolytic drugs that have been studied and which have demonstrated cytotoxic effects in treating different cancer cell lines.

Category	Name of the Drug	Mechanism of Action	In Vivo or In Vitro Studies, Type of Cancer
Targeting glucose transporters	GLUT-1 antibody	Inhibits GLUT 1	Inhibits proliferation and induces apoptosis in NSCL and breast cancer [24].
	Ritonavir	Inhibits Protease and GLUT 4	Has shown a cytotoxic effect on multiple myeloma, breast, ovarian, and melanoma cancer cell lines [25].
	BAY-876	GLUT 1 receptor antagonist	Can be effective in HCC [26].
	Fasentin	GLUT 1 receptor antagonist	Limited data are available.
	Phloretin	Blocks GLUT 2	Induces apoptosis in HCC and suppresses the protein kinase C pathway in melanoma cell lines [27,28].
	Silybin	Blocks GLUT 4	Inhibits growth in HCC cell lines [29].
	Quercetin	Blocks GLUT 1	An increase in the BAX/BCL-2 ratio induces apoptosis in HCC cell lines [30].
	Canagliflozin	SGLT2 inhibitor	Reduced growth of liver cancer cells [31].
Targeting enzymes involved in glycolysis	2-deoxy-D-glucose	Inhibits hexokinase inhibitor	Cellular ATP depletion and apoptosis in HepG2 and Hep3B HCC cell lines [32,33]. Evaluated in prostate cancer, glioma blastoma multiforme, and anaplastic astrocytoma [34,35,36].
	Dichloroacetate	Inhibits pyruvate dehydrogenase kinase	Redirecting cellular metabolism and reversing the Warburg effect in different tumor cells, including the breast, prostate, medullary thyroid, lung, myeloma, endometrial, and glioblastoma multiforme [37,38,39,40,41,42].
	3-Bromopyruvate	Inhibits GAPDH and hexokinase	Effectively suppresses glycolysis and impedes ATP production, leading to apoptosis. Both in vitro and in vivo showed antitumor effects on HCC [43,44].
	Bumetanide	Inhibits carbonic anhydrase IX (CAIX) and XII (CAXII)	Lactic acid accumulation and decrease in cellular pH. Studies in an N1S1 HCC tumor model in rats have been shown to sever tumor necrosis [45].

GLUT = Glucose transporter; NSCL = non-small cell lung; HCC = Hepatocellular carcinoma.

## Data Availability

Not applicable.
